# Neuro-Behavioral Correlates of Executive Dysfunctions in Dyslexia Over Development From Childhood to Adulthood

**DOI:** 10.3389/fpsyg.2021.708863

**Published:** 2021-08-23

**Authors:** Rola Farah, Silvio Ionta, Tzipi Horowitz-Kraus

**Affiliations:** ^1^Educational Neuroimaging Center, Faculty of Education in Science and Technology, Technion, Haifa, Israel; ^2^Reading and Literacy Discovery Center and the Pediatric Neuroimaging Research Center, Cincinnati Children’s Hospital Medical Center, Cincinnati, OH, United States; ^3^Sensory-Motor Lab (SeMoLa), Department of Ophthalmology, University of Lausanne, Lausanne, Switzerland; ^4^Jules Gonin Eye Hospital-Fondation Asile des Aveugles, Lausanne, Switzerland

**Keywords:** development, dyslexia, executive function, language, reading, neuroimaging

## Abstract

Dyslexia is a neurobiological learning disability in the reading domain that has symptoms in early childhood and persists throughout life. Individuals with dyslexia experience difficulties in academia and cognitive and emotional challenges that can affect wellbeing. Early intervention is critical to minimize the long-term difficulties of these individuals. However, the behavioral and neural correlates which predict dyslexia are challenging to depict before reading is acquired. One of the precursors for language and reading acquisition is executive functions (EF). The present review aims to highlight the current atypicality found in individuals with dyslexia in the domain of EF using behavioral measures, brain mapping, functional connectivity, and diffusion tensor imaging along development. Individuals with dyslexia show EF abnormalities in both behavioral and neurobiological domains, starting in early childhood that persist into adulthood. EF impairment precedes reading disability, therefore adding an EF assessment to the neuropsychological testing is recommended for early intervention. EF training should also be considered for the most comprehensive outcomes.

## Introduction

### Dyslexia: Definition and Characteristics

Since its first description over a century ago (Morgan, 1896), dyslexia has been investigated by cognitive and neurobiological studies to better understand the underlying mechanisms. One of the models that aim to describe the reading process in an attempt to explain the possible underlying impaired mechanisms in dyslexia is the simple view of reading model (Adlof et al., 2006). This model claims that reading comprehension is achieved by a combination of both language processing abilities and word decoding with EF abilities added to the model in recent years (Cutting et al., 2015). The present review summarizes the available neuro-behavioral evidence about such mechanisms to provide a compelling picture of dyslexia, the associated neuro-behavioral aspects, and possible input for intervention while focusing on the EF deficit in dyslexia.

Dyslexia is a neurobiological learning disability, affecting 5–17% of the population (Gabrieli, 2009) and is defined by word recognition difficulty and poor spelling abilities despite normal intelligence and adequate education and exposure to written material (Shaywitz and Shaywitz, 2008). Characteristics include inaccurate word recognition and decoding along with difficulties in reading comprehension (Directors of IDA, 2002). Word reading and reading comprehension have been found to be similar constructs; however, reading comprehension has been related to working memory abilities, and speed of processing was found to be a specific predictor for better word reading (Christopher et al., 2012).

Learning to read involves several critical steps: directing visual and auditory attention to the written stimuli and avoiding distractors, decoding of the word (i.e., phonological processing), visually perceiving the word and corresponding it to sound and receiving semantic information about the word (Horowitz-Kraus, 2016). Moving between the different steps demands mental resources crucial for successful reading, such as inhibition, working memory, shifting, and speed of processing (Booth et al., 2014). Therefore, it is not surprising that several theories have been raised to explain the underlying causes for reading deficits in dyslexia. The *phonological deficit theory* suggests that a dysfunction in the peri-sylvian region could lead to poor phonological skills in dyslexia (e.g., Ramus, 2003; Vellutino et al., 2004). This theory claims that the primary deficit in these readers might be the inability to translate written graphemes into their corresponding sounds due to a basic impairment in their phonological processor. An extension for this theory is called the “*double deficit theory*” suggesting a deficit not only in sound decoding and letter-sound matching but also in naming (letters, objects, etc.; Wolf and Bowers, 2000). The *orthographical deficit theory* postulates that dyslexia could stem from deficient orthographic imagery processing; these individuals suffer from an inability to perceive words holistically, leading to challenges in word recognition and the comprehension of orthographic information (Badian, 2005). This, in turn, leads them to an inability to establish a sufficient mental lexicon (i.e., self-teaching hypothesis; Share and Shalev, 2004). The *morphological deficit theory* suggests that dyslexia comes from poor knowledge of morphemes, decreasing written fluency (Nagy et al., 2006). The *asynchrony theory* (Breznitz, 2006) states that the core cause for dyslexia is a speed of processing deficit during word decoding. The *magnocellular deficit theory* has also been proposed, hypothesizing that the foundation of dyslexia arises from a dysfunction of the magnocellular visual system, causing a dysfunction in the processing of speedy temporal information (Stein and Walsh, 1997). The *cerebellar deficit theory* points at the dysfunction of the cerebellum in automatic word recognition (Nicolson et al., 2001). Lastly, a *temporal processing deficit theory* has been proposed, in that dyslexia would stem from a difficulty in fast temporal processing, most specifically in the low-level auditory domain (Tallal, 1980). More recently, the spread of modern neuro-investigation techniques to study dyslexia, the introduction of innovative experimental protocols, and the implementation of advanced data analyses brought to light the theory that the symptoms of dyslexia could result from impaired executive functioning (EF; Helland and Asbjørnsen, 2000; Brosnan et al., 2002; Reiter et al., 2005; Berninger et al., 2006; Smith-Spark and Fisk, 2007; Altemeier et al., 2008; Horowitz-Kraus, 2014; Varvara et al., 2014; Butterfuss and Kendeou, 2017).

### Executive Functions and Dyslexia

EFs is a broad term for top-down cognitive processes that aid in creating, planning, performing, and achieving goals (Lezak, 1982; Miyake et al., 2000). Three “main” EFs have been found *via* factor analyses: inhibition, shifting, and updating (Miyake et al., 2000) with others building upon Miyake and colleagues work, suggesting the inclusion of working memory and flexibility as well (Diamond, 2013). Inhibition is defined as the ability to impede automatic responses when necessary and can be tested by the Stroop (1935), anti-saccade (Hallett, 1978), and stop-signal tasks (Logan, 1994). Details regarding the sub-processes of inhibition during these paradigms can be found in Kok (1999). Shifting refers to switching between multiple tasks (Monsell, 1996) and can be assessed using the Wisconsin Card Sorting Task (Nyhus and Barceló, 2009), the Trail-Making Task Test B (Arbuthnott and Frank, 2000), and category switch test (Friedman et al., 2008). These functions are distinct yet rely on one another (Miyake et al., 2000). Other factor analyses on EFs and children point predominantly to working memory and shifting (Lehto et al., 2003; Huizinga et al., 2006) and processing speed (Span et al., 2004; Anderson and Reidy, 2012). A full list of tasks assessing different EF domains (i.e., updating, working memory, inhibition, shifting, short-term memory, and speed of processing) can be found in Friedman et al. (2008), Anderson and Reidy (2012), and Butterfuss and Kendeou (2017).

The development of EF is tightly connected to reading development. A theoretical review by Butterfuss and Kendeou (2017) demonstrated how EF is “embedded” within various reading models, including the construction-integration model (Kintsch, 1988), the structure-building framework (Gernsbacher, 1991), the resonance model (Albrecht and O’Brien, 1993), the event-indexing model (Zwaan et al., 1995), the casual network model (Trabasso et al., 1989), the constructionist theory (Graesser et al., 1994), and the landscape model (Van den Broek et al., 1999). The construction-integration model (Kintsch, 1988) involves related information links that assist each other and irrelevant information links that inhibit each other, relying on the EF inhibition. The structure-building framework (Gernsbacher, 1991) suppresses irrelevant information that does not correspond with the current structure. The resonance model (Albrecht and O’Brien, 1993) found that phrases related to the text strengthened the target, whereas irrelevant phrases were suppressed. The event-indexing model (Zwaan et al., 1995) involves shifting from one dimension (i.e., time, space, causality, motivation, and agents) to another. The casual network model (Trabasso et al., 1989) includes memory for narrative elements (i.e., settings, events, goals, attempts, outcomes, and reactions). The constructionist theory (Graesser et al., 1994) contains cognitive control mechanisms in the search for semantics, likely shifting. Even higher-level EFs, such as planning, have also been hypothesized to facilitate reading (Kendeou et al., 2015). These models highlight that EF plays a crucial role in reading. However, it is important to note that not one single model can fully explain the heterogeneous patterns in both reading and EF abilities found in individuals with dyslexia.

Unlike language development abilities that reach their peak in early childhood (age 7; Purves et al., 2001), EF may mature during adulthood (mid-twenties; Romine and Reynolds, 2005). Therefore, to better understand how academic achievements, especially reading, rely on EF, it is important to discuss the time each of these abilities develops in life. Some components of EF develop relatively early along development and some mature later on until a full maturation of the prefrontal cortex at the age of 25 (Giedd et al., 2009). Inhibition has been shown to develop at 12 months (Diamond and Goldman-Rakic, 1989), reaching adult levels at age 12 years (Welsh et al., 1991). Planning skills have been found to fully mature between 9 to 13 years old (Welsh et al., 1991; Anderson et al., 1996). Shifting begins to occur around 4 to 5 years (Espy, 1997) and increases significantly at 7 years old (Anderson, 2002) and updating, which requires monitoring and coding of information in memory and is related to verbal and visuospatial working memory abilities (St Clair-Thompson and Gathercole, 2005). Therefore, not all EFs may follow the same developmental trend (Passler et al., 1985; Lehto et al., 2003; Jurado and Rosselli, 2007), and some studies have found later ages of EF maturation (Huizinga et al., 2006) or that processing speed could be the driving factor of EF maturation in children (Span et al., 2004). A longitudinal study done by Altemeier et al. (2008) found that all EFs may not develop the same in typical readers grades 1 to 6: inhibition abilities can increase consistently, whereas rapid automatic switching and combined inhibition and switching may begin to slow in the rate along development around fourth grade. However, another theory suggested by Miyake and Friedman (the unity/diversity framework) claims that EF (including updating, shifting, and inhibition) is relatively stable along development (Miyake and Friedman, 2012).

In sum, although reading acquisition is formally acquired at the age of 6 and EF fully matures later in life, these abilities are tangled in the reading process. A crucial question arises as to the involvement of EF in the atypical course of reading development (i.e., in dyslexia), using behavioral and neurobiological measures. Of note, dyslexia often co-occurs with attention deficit hyperactivity disorder, comorbidity characterized by EF deficit (Pennington et al., 1993; Willcutt et al., 2001, 2005). However, as the current review does not focus on comorbidities, it includes studies focusing only on dyslexia and the involvement of EF in this disorder, focusing on the developmental model of sub-components of EF, based on Anderson’s approach (Anderson and Reidy, 2012). This model was chosen as it relates to the development of several sub-components of EF (not limited to the three core components), as well as to the attention system from birth onwards, which provides a longitudinal framework to this review focusing on the involvement of EF in dyslexia along life span.

## Materials and Methods

### Searching the Relevant Papers

PubMed and Google Scholar were used to search for studies exploring behavioral and neurobiological dysfunction in dyslexia. The following keywords were used as: “executive function,” “cognitive control,” “functional MRI,” “structural MRI,” “EEG,” “reading,” and “dyslexia” as well as the combinations including the cognitive abilities/dyslexia and neuroimaging methods. This search generated over 200,000 manuscripts with 171,000 manuscripts for executive functions/cognitive control and “dyslexia” and approximately 38,000 for “dyslexia” and functional/structural “MRI” or “EEG.” The studies reporting of evidence about neurobiological changes in dyslexia in relation to EF were included in this nonsystematic review. The brain regions per developmental group were defined based on the automated anatomical labeling (AAL) atlas (Tzourio-Mazoyer et al., 2002). AAL is a software commonly implemented within neuroimaging analysis tools, such as Statistical Parametric Mapping to identify the brain regions comprised within specific neural activation blobs according to a standard brain atlas (for more information see Rolls et al., 2020). All images were created using the BrainNet Viewer (Xia et al., 2013).

## Results

Individuals with dyslexia demonstrate deficits in EFs, with a varied profile along development (see Table 1). Children at risk for dyslexia may demonstrate more challenges in selective attention and visuospatial short-term memory. Children show more deficits in planning, teenagers show more deficits in speed of processing, and adults show difficulties in planning and speed of processing. All age groups show deficits in working memory. One possibility for these changes along age is the gradual maturation and increased connections within the frontal lobe and between the frontal lobe and other brain regions (Fair et al., 2009).

**Table 1:** Executive dysfunctions in individuals with dyslexia along development.

Executive function	Children at risk (0–5)	Children with dyslexia (6–12)	Teenagers with dyslexia (13–21)	Adults with dyslexia (22+)
Inhibition	Gooch et al., 2014	Lazarus et al., 1984; Kelly et al., 1989; Everatt et al., 1997; Helland and Asbjørnsen, 2000; Carretti et al., 2005; Reiter et al., 2005		
Memory	Gooch et al., 2014	Fein et al., 1988; Barnea et al., 1994; Chiappe et al., 2000; Brosnan et al., 2002; Reiter et al., 2005; Berninger et al., 2006	Fein et al., 1988; Chiappe et al., 2000; Brosnan et al., 2002; Carretti et al., 2005; Horowitz-Kraus, 2015	Fein et al., 1988; Chiappe et al., 2000; Brosnan et al., 2002; Berninger et al., 2006; Horowitz-Kraus and Breznitz, 2009
Shifting		Kelly et al., 1989; Kershner and Morton, 1990; Helland and Asbjørnsen, 2000; Horowitz-Kraus, 2014	Asbjørnsen and Bryden, 1998; Kraus and Horowitz-Kraus, 2014; Horowitz-Kraus, 2015	
Speed of processing			Horowitz-Kraus, 2015	Breznitz and Misra, 2003; Horowitz-Kraus and Breznitz, 2011
Attention	Facoetti et al., 2010; Gooch et al., 2014; Hardeman, 2016	Facoetti et al., 2000		
Problem solving and planning		Chiarenza, 1990; Levin, 1990; Mati-Zissi et al., 1998; Reiter et al., 2005		Catts, 1989; Weyandt et al., 1998

### Behavioral Evidence

#### Executive Dysfunctions in Individuals With Dyslexia

EFs develop along life span (Diamond, 1985; Altemeier et al., 2008). Additionally, in individuals with reading difficulties, some longitudinal studies reported deficits in working memory observed from age 6 to 49 years (Chiappe et al., 2000). As explained, due to brain maturation differences, especially in relatedness to EF, the deficit in EFs among individuals with dyslexia in 3 age groups will be reviewed: children “at-risk” for dyslexia (ages 0–5 years), children (ages 6–12 years), adolescents (ages 13–21), and adults (ages 22 and up), both behaviorally and neurobiologically.

#### Executive Dysfunctions in Children at Risk for Dyslexia (0–5 Years)

Children at risk for dyslexia (i.e., with parents or siblings with dyslexia), between the ages of 3 to 5 years, show EF impairment, in the domains of selective and sustained attention, inhibition, and visuospatial short-term memory which were found to be correlated with language ability (Gooch et al., 2014). Executive functions play a role as a predictor of future reading disability at 4.5 years old in a population at risk (Thompson et al., 2015). Three-year-olds at risk of dyslexia exhibited a trend of lower scores on selective attention. However, the effects of inhibition or working memory were not significant (Hardeman, 2016). Overall, the literature related to children at risk for dyslexia before reading age points at attention, inhibition, and visuospatial short-term memory as EF components which may be “precursors” for dyslexia before reading age.

#### Executive Dysfunctions in Children With Dyslexia (6–12 Years)

Children with dyslexia (6–12 years old) have shown impairment in inhibition, as assessed by the Stroop task (Lazarus et al., 1984; Kelly et al., 1989; Everatt et al., 1997; Helland and Asbjørnsen, 2000; Reiter et al., 2005), as well as in verbal and nonverbal working memory (Fein et al., 1988; Barnea et al., 1994; Brosnan et al., 2002; Reiter et al., 2005; Berninger et al., 2006). Children with dyslexia ages 6–12 years also show difficulties with planning (Chiarenza, 1990; Levin, 1990; Mati-Zissi et al., 1998), as shown by worse performance on the Tower of London (Reiter et al., 2005). Children with dyslexia ages 11 and 12 display complications on shifting (Kelly et al., 1989; Kershner and Morton, 1990; Helland and Asbjørnsen, 2000), also shown through more errors and a slower reaction time when performing the Wisconsin Card Sorting Test (Horowitz-Kraus, 2014). Carretti et al. (2005) found that poor readers made more intrusion errors, supporting that working memory may aid reading comprehension through inhibition in children. Related to the visual attention difficulties suggested in adults (Smith-Spark and Fisk, 2007), 8–17-year-old children with reading difficulties showed deceased visual and auditory spatial attention difficulties which were also related to their decreased reading abilities (Varvara et al., 2014). By that, the authors concluded that these readers showed a deficiency in their central executive system (Varvara et al., 2014). These results were also observed by others and extended to switching/shifting abilities as measured using the Wisconsin task in children ages 8–17 years old with dyslexia (Menghini et al., 2010). Overall, research on beginning readers points to inhibition, visual/auditory attention, and working memory dysfunction, along with planning and shifting challenges in 6–12-year-old children as altered EF in children with dyslexia compared to age-matched typical readers.

#### Executive Dysfunction in Teenagers With Dyslexia (Ages 13–21 Years)

Teenagers with dyslexia ages 13–21 years old exhibited deficits in EFs, specifically in the domains of verbal and nonverbal working memory (Fein et al., 1988; Brosnan et al., 2002; Horowitz-Kraus, 2015), shifting (Asbjørnsen and Bryden, 1998), and speed of processing (Horowitz-Kraus, 2015). In other studies, readers with dyslexia also showed deficits in error detection during the Madrid Card Sorting Task, similar to the Wisconsin Card Sorting Task assessing shifting/switching, with slower reaction times and more errors (Kraus and Horowitz-Kraus, 2014; Horowitz-Kraus, 2015). These readers also showed impairment in error monitoring in reading tasks as well (Horowitz-Kraus, 2011). In general, research highlights difficulties both in more basic EF as well as more complex EFs, such as in the domains of working memory, shifting, speed of processing, and also error detection and monitoring for teenagers with dyslexia.

#### Executive Dysfunction in Adults With Dyslexia (22 Years and Older)

Adults with dyslexia have shown difficulties with planning (Catts, 1989; Weyandt et al., 1998), working memory (Fein et al., 1988; Brosnan et al., 2002; Berninger et al., 2006; Horowitz-Kraus and Breznitz, 2009), and visual processing (visual-spatial working memory) abilities (Smith-Spark et al., 2016; Provazza et al., 2019). College students with dyslexia have shown impairments in speed of processing (Breznitz and Misra, 2003; Horowitz-Kraus and Breznitz, 2011), which were related to their reading accuracy and reaction time. Brosnan’s significant differences between adults with dyslexia and typical readers were found in the EF domains of planning, sequencing, and organization of memory and visual-spatial measures (Brosnan et al., 2002). However, Smith-Spark and colleagues suggested that the difficulties in working memory were extended into visual-spatial attention abilities in adulthood, which support a central difficulty in EF in this population (Smith-Spark et al., 2003; Smith-Spark and Fisk, 2007). These findings were echoed by Provazza and colleagues demonstrating a similar visual processing challenge in this population (Provazza et al., 2019). Considering the brain networks involved, for example, in visuospatial processing (Perruchoud et al., 2016) or attention (Pamplona et al., 2020) comprise a large number of brain regions and related interconnections both in adulthood and development (Ionta, 2021), it is possible that in individuals with dyslexia, possibly over time/age, natural neuroplastic compensatory mechanisms are put in place to establish alternative neural activations/connections which would make dyslectic people able to compensate their deficits and resemble the performance of their age-matched non-dyslectic peers in the domains of organization, visual-spatial abilities, shifting, and attention.

Despite the great benefit behavioral and cognitive testing provide when discussing reading and EF abilities in those with dyslexia, one limitation of behavioral tests is that they can be considered versatile in the functions they assess. As there is a current debate in the literature of the definition and assessment of EFs, the current strength of validity in EF assessment should be taken with some caution (Jurado and Rosselli, 2007). However, the behavioral tests used in this review have been used for many years and in many studies in assessing EFs (Axelrod et al., 1994; Romine et al., 2004; Carlson, 2005). Neuroimaging data can assist with this limitation, by giving the ability to differentiate network functionality. As different networks have been attributed to more basic attention abilities and to higher-level monitoring, different aspects of EFs can be separated and assessed using neuroimaging techniques (Dosenbach et al., 2008; Petersen and Posner, 2012).

### The Neurobiology of Executive Function in Dyslexia

EFs seem to be important in the reading process, as EF areas (i.e., the inferior frontal gyrus, middle frontal gyrus, precuneus, and posterior cingulate) are also involved during reading-related tasks in individuals from 5 to 18 years (Karunanayaka et al., 2007) as well as in a listening-doing matching system (Halje et al., 2015). Additionally, since EFs rely on multiple brain regions, they may be particularly sensitive to brain dysfunction (Reiter et al., 2005). Brain regions associated with EFs have also been found to be correlated with reading ability. Greater functional connectivity of an EF network (i.e., the cingulo-opercular network) has been found, accompanying gains in both reading and EF behavioral measures after a reading intervention (Horowitz-Kraus et al., 2015). Greater connectivity between EF and visual regions has also been correlated with greater reading comprehension post-reading intervention (Horowitz-Kraus et al., 2014). Overall, the scientific literature provides evidence for the importance of neurobiological EF measures for reading. Here, we review the neurobiology of dyslexia in a developmental manner, focusing on EF networks during reading-related tasks.

#### Neural Circuits Related to EF in Children at Risk for Dyslexia (0–5 Years)

Most of the current neuroimaging research done with infants and young children at risk for dyslexia is with electroencephalography (EEG) and event-related potentials (ERPs). When compared to the behavioral performance of a head-turn task, smaller bilateral response in at risk infants was associated with greater performance, whereas in controls, greater left hemisphere response was associated with greater performance, suggesting that at risk infants may have differential neural processes involved for auditory and language tasks already at 6 months (Lyytinen et al., 2004). Zuijen et al. (2013) found that 2-month-olds at risk for dyslexia who later at age 7 years scored poorly on a word reading fluency measure did not show a mismatch response, whereas infants at risk for dyslexia and controls who later performed well on a fluency measure did show a mismatch response, showing differentiation of processing to two different auditory sounds (Zuijen et al., 2013). These aberrant event-related responses are also found in 6-month-olds at risk for dyslexia (Leppänen et al., 2002) and *via* delayed P100 [representing selective attention (Mangun and Hillyard, 1991)] and P200 [associated with working memory and attention (Lijffijt et al., 2009)] peaks for standard auditory stimuli in children at risk for dyslexia (van Herten et al., 2008). The N200 response [an ERP related to inhibition (Heil et al., 2000)] is absent in at risk 2-year-olds compared to controls during lexical-semantic priming (von Koss Torkildsen et al., 2007).

**Figure 1:**
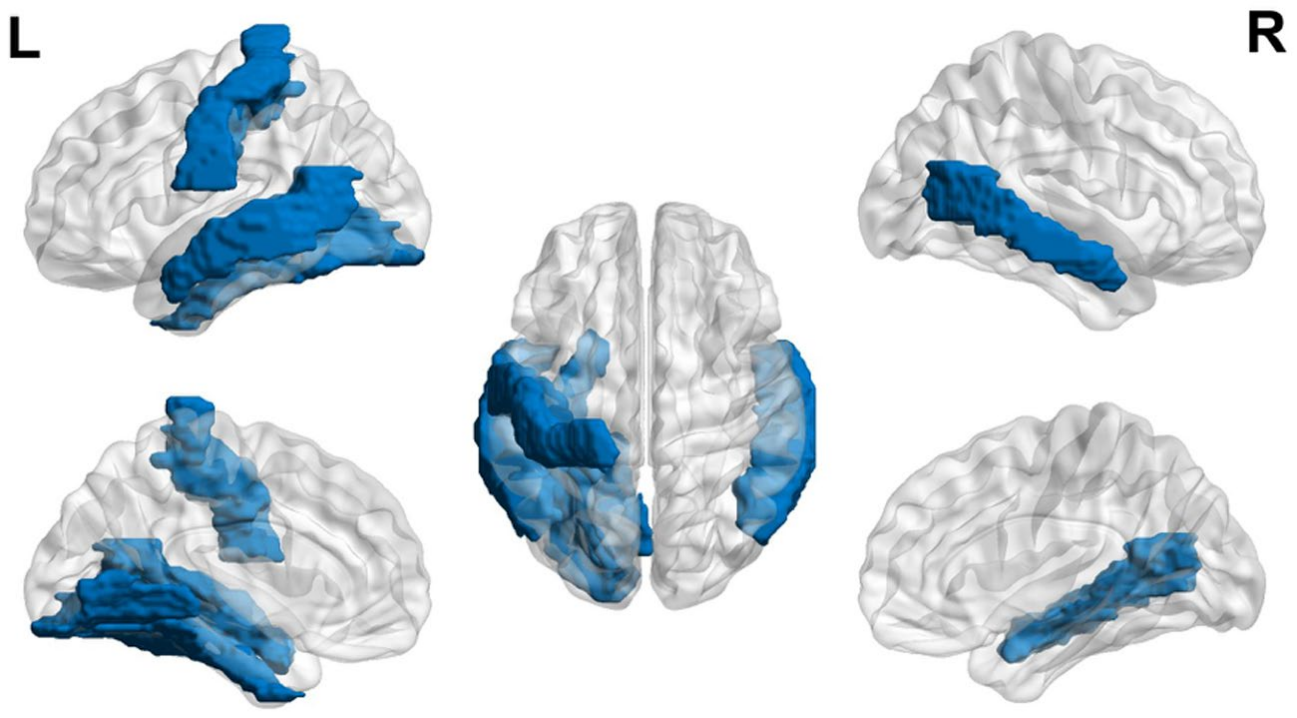
Neural circuit dysfunction related to reading circuits in infants at risk (ages 3–5) with dyslexia. Decreased activation in bilateral middle temporal gyrus, left lingual gyrus, left postcentral gyrus, and left fusiform gyrus (Raschle et al., 2012) related to EF in children ages 3–5 at risk for dyslexia. The blue color represents decreased activation. Data are presented in sagittal and axial views in the left and right hemispheres (L = left and R = right) over a glass brain. Regions were defined based on Automated Anatomical Labeling (AAL) atlas (Tzourio-Mazoyer et al., 2002). Image created using the BrainNet Viewer (Xia et al., 2013).

Using functional MRI in 5-year-old children at risk for dyslexia, children were asked to listen to two words and decide if they both started with the same beginning sound (Raschle et al., 2012). Children at risk for dyslexia exhibited hypoactivation in bilateral occipito-temporal and left temporo-parietal regions (Raschle et al., 2012; see Figure 1). This finding corresponds to a decrease in gray matter found in the left occipito-temporal, bilateral parieto-temporal, left fusiform gyrus, and right lingual gyrus (Raschle et al., 2011). In as young as 6 to 18 months, Langer et al. (2017) found lower fractional anisotropy in the left arcuate fasciculus. Children ages 3–5 years showed increased functional connectivity of their future reading network and language processing regions and regions influencing EFs (i.e., left Brodmann area 2, 13, and 44 and right Brodmann area 6 and 44) for greater maternal fluency ability (Horowitz-Kraus et al., 2017).

#### Neural Circuits Related to EF in Children With Dyslexia (6–12 Years)

Using EEG, Duffy et al. (1980) found higher alpha values in children with dyslexia in the bilateral medial frontal region during a naming abstract figures task and a reading task and only in the left medial frontal region during speech, a sound-symbol-association test, and during rest with eyes open (Duffy et al., 1980). Higher alpha values were also observed during a Kimura figures test (assessing nonverbal memory) in the left anterolateral frontal region (Duffy et al., 1980). The authors suggest this may represent a hypoactivation of frontal systems (Duffy et al., 1980), conflicting with the hyperactivation seen in frontal networks in fMRI studies.

Some studies have shown that children with dyslexia may display neurobiological dysfunction related to EF as well as language systems during reading tasks. During a narrative comprehension task, hyperactivation in the right superior frontal gyrus and right middle frontal gyrus, both areas involved in EF, was observed in children with dyslexia (Horowitz-Kraus et al., 2016). During sentence comprehension, children with dyslexia exhibited hyperactivation in the left middle/superior temporal gyri and bilateral insula [part of the cingulo-opercular network (Power et al., 2011)], right cingulate gyrus [also in the cingulo-opercular network (Power et al., 2011)], right superior frontal gyrus [an area involved in working memory tasks (Johnson et al., 2003)], and the right parietal lobe (Rimrodt et al., 2008). During a reading task, children with dyslexia displayed hypoactivation in phonological areas, such as the left fusiform gyrus (visual word form area) and Wernicke’s area, whereas hyperactivation was seen in bilateral orthographic areas (i.e., anterior visual word form areas and posterior bilateral middle temporal gyri; Saralegui et al., 2014). The authors hypothesize that individuals with dyslexia compensate for phonological deficits by hyper activating areas in the orthographic route.

Seki et al. (2001) also found compensatory activation during a reading task in the bilateral occipital cortex, inferior frontal regions (areas involved in EF), and inferior precentral gyrus. The involvement of visual and EF-related regions was also observed during a phonological task, one of the basic abilities impaired in dyslexia: hyperactivation of the left inferior frontal gyrus (an area involved in EF; Georgiewa et al., 2002) and hypoactivation of the right visual and left occipital cortex (Shaywitz et al., 2002; Liu et al., 2012) and temporal and prefrontal cortices (involved in EF; Backes et al., 2002; Shaywitz et al., 2002), specifically the left superior temporal gyrus (Kita et al., 2013), the left fusiform cortex (Desroches et al., 2010), and left inferior frontal gyrus (an area involved in EF; Liu et al., 2012).

Interestingly, also subcortical regions were found to show hypoactivation during a phonological processing task in children with dyslexia, such as the basal ganglia (Kita et al., 2013), in addition to the left extrastriate cortex (Backes et al., 2002) and the right cerebellum (van Ermingen-Marbach et al., 2013). Considering the importance of mutual exchanges between cortical and subcortical regions (Zeugin and Ionta, 2021), these findings demonstrate how more basic learning mechanisms related to cortico-subcortical-cerebellar activations are also different in children with dyslexia. It has been proposed that this hyperactivation in the above-mentioned EF regions and hypoactivation of visual processing and language-related regions is related to the pathology of dyslexia, through a greater attempt of recognizing words holistically and retrieving the semantic meaning of it from working memory and allocating greater attention for error detection to compensate for hypoactivation in visual and reading-related areas.

Shaywitz et al. (2002) found greater activation in bilateral inferior frontal gyri (areas involved in EF) in older children. These results can be viewed in a network-based framework: higher global efficiency in the fronto-parietal network was negatively correlated with cognitive tests on narrative comprehension, phonological awareness, word and non-word reading, and executive abilities (assessed *via* the Stroop task; Horowitz-Kraus et al., 2016). When analyzed longitudinally, an upregulation of connectivity in occipito-temporal connections and a downregulation in inferior frontal gyri connections was found from 6 to 8 years, but from 8 to 12 years connectivity was similar to controls. This finding supports the hypothesis that abnormalities in the EF network could precede dysfunction in the reading network (Clark et al., 2014). Overall, increased right hemisphere connectivity has been found in children with dyslexia (Finn et al., 2014).

Extending the functional changes in EF and visual related regions also to the structural domain in children with dyslexia in this age group was suggested by Williams et al. (2017) who found thinner cortex in bilateral occipito-parietal and inferior temporal cortices (i.e., portions of the reading network) compared to controls (Williams et al., 2017). Thinner cortex was also found in areas relevant for cognitive control (i.e., the right orbitofrontal, left anterior cingulate, left superior parietal, and right medial parietal cortices). The orbitofrontal cortex is involved in learning from probabilistic feedback (Tsuchida et al., 2010) and in decision making (Kringelbach, 2005). This finding could explain why lower error-related negativities (i.e., a neural mechanism that is activated when an error is made) and a higher error rate have been found in dyslexics compared to controls (Horowitz-Kraus, 2012). Support for the structural alterations in EF and visual regions in children with dyslexia is provided by diffusion tensor imaging studies. Slight fractional anisotropy (FA) decreases have also been found in left temporo-parietal neural pathways in children with dyslexia (Deutsch et al., 2005). Further, children with dyslexia showed reduced FA in the left superior longitudinal fasciculus, connecting the frontal and parietal lobes (Carter et al., 2009; Rollins et al., 2009), along with the left corona radiata, the left centrum semiovale (Odegard et al., 2009), the left inferior frontal gyrus (an area involved in EF), and temporo-parietal areas (Rimrodt et al., 2010). Corpus callosum differences are also seen, with a smaller genu that runs along the anterior cingulate cortex (related to EF) in dyslexic children (Hynd et al., 1995).

**Figure 2:**
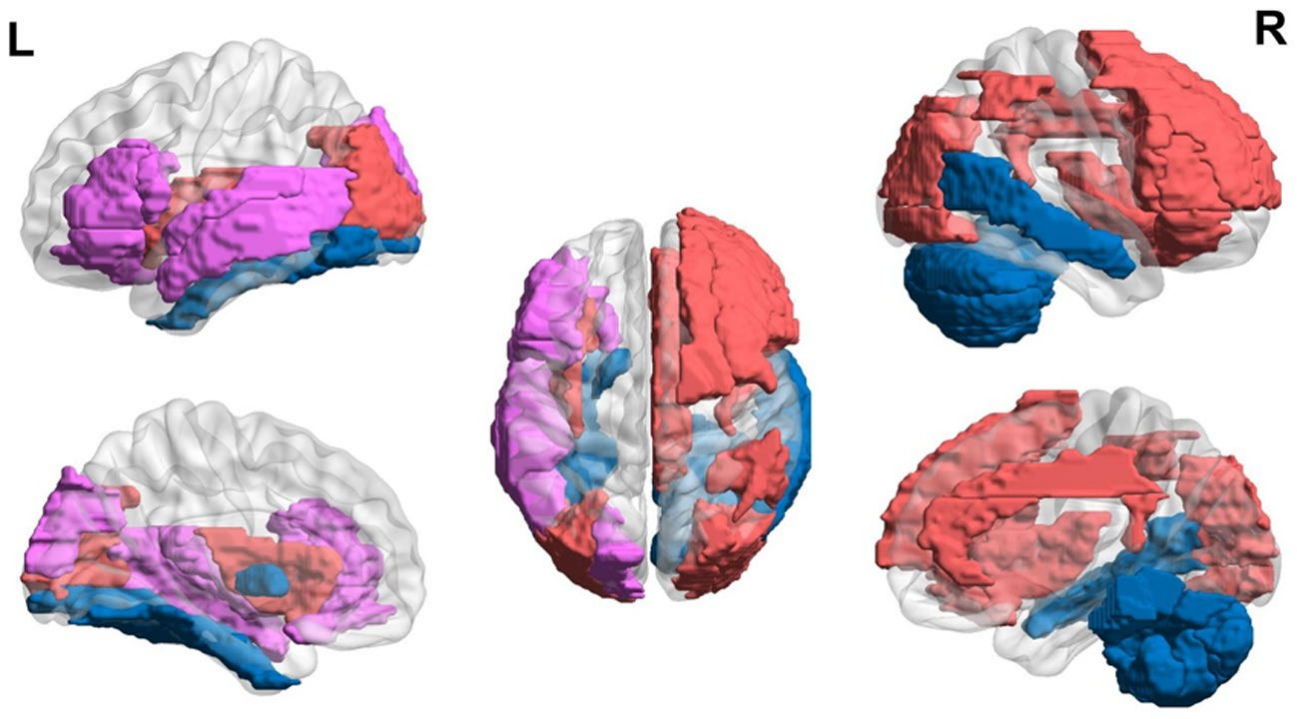
Neural circuit dysfunction related to reading circuits in children (ages 6–12) with dyslexia. Decreased activation in left superior temporal gyrus (Kita et al., 2013; Saralegui et al., 2014), bilateral middle temporal gyrus (Saralegui et al., 2014), left fusiform gyrus (Desroches et al., 2010; Saralegui et al., 2014), left globus pallidus (Kita et al., 2013), left inferior frontal gyrus (Liu et al., 2012), right cerebellum (van Ermingen-Marbach et al., 2013), and left superior occipital gyrus (Backes et al., 2002; Shaywitz et al., 2002) and increased activation in the right superior frontal gyrus (Horowitz-Kraus et al., 2016), right middle frontal gyrus (Horowitz-Kraus et al., 2016), bilateral insula (Rimrodt et al., 2008), left superior temporal gyrus (Rimrodt et al., 2008), left middle temporal gyrus (Rimrodt et al., 2008), right cingulum (Rimrodt et al., 2008), right inferior parietal lobule (Rimrodt et al., 2008), bilateral occipital gyrus (Seki et al., 2001), and bilateral inferior frontal gyrus (Seki et al., 2001; Georgiewa et al., 2002; Shaywitz et al., 2002), related to EF in children ages 6–12 at risk for dyslexia. Red represents areas of hyperactivity, blue areas of hypoactivity, and purple areas where papers have reported hyperactivity and hypoactivity. Data are presented in sagittal and axial views in the left and right hemispheres (L = left and R = right) over a glass brain. Regions were defined based on AAL atlas (Tzourio-Mazoyer et al., 2002). Image created using the BrainNet Viewer (Xia et al., 2013).

Overall, the literature suggests that children with dyslexia ages 6 to 12 years demonstrate hyperactivation in areas related to EF and hypoactivation in areas related to language and visual areas during reading-related tasks using MRI (see Figure 2). Nine- and 10-year-olds demonstrate increased alpha in frontal areas during reading-related tasks. Structural differences are also found with the thinner cortex in reading-related and EF areas.

#### Neural Circuits Related to EF in Teenagers With Dyslexia (13–21 Years)

As individuals with dyslexia reach teenage years, dysfunction has been found in EF circuits during reading tasks as well, with reports showing changes in brain responses during the Wisconsin shifting/swathing task (decreased feedback-related negativity, i.e., an ERP related to brain activation associated with a response to feedback and is part the monitoring system) in children ages 12–14 years with dyslexia vs. typical readers (Kraus and Horowitz-Kraus, 2014).

When assessing teenagers with dyslexia versus age- and reading-matched typical readers during a visual word rhyme judgment task (assessing the phonological analysis of orthographic input), hyperactivation was found in EF areas (i.e., the left inferior and middle frontal gyri and caudate) and the thalamus compared to age-matched controls (Hoeft et al., 2007). However, additional support for alteration related to visual processing was observed by hypoactivation in left parietal and fusiform regions with both age- and reading-matched controls (Hoeft et al., 2007). However, hyperactivation has also been found in visual areas (Wimmer et al., 2010). Hypoactivation seen in dyslexia could then be attributed to a malfunction in “classic” dyslexic posterior malfunction, whereas the hyperactivation could be a compensatory response in EF areas to offset the dysfunction found in posterior areas (Hoeft et al., 2007). Desroches et al. (2010) also found hypoactivation of the left fusiform cortex during a phonology task. During a phonological task, hypoactivation in the posterior regions of the reading network (Brambati et al., 2006), in a left ventral occipito-temporal region, a left inferior parietal region, and a left inferior frontal region [an area related to EF (Wimmer et al., 2010; Steinbrink et al., 2012)] was observed. During an N-back task, children with dyslexia displayed hypoactivation in the left superior parietal lobule and the right inferior prefrontal gyrus (Beneventi et al., 2010). During the Madrid Card Sorting Task, teenagers with dyslexia showed decreased target-locked N100, involved in selective attention (Hillyard et al., 1973), and P300, also involved in attention (Polich, 1986) and amplitudes (Kraus and Horowitz-Kraus, 2014; Horowitz-Kraus, 2015).

An altered participation of EF during phonological processing was observed *via* functional connectivity between phonological processing-related regions and an EF network (Wolf et al., 2010). A left prefrontal network exhibited increased connectivity in the left prefrontal and inferior parietal regions (Wolf et al., 2010). A bilateral executive fronto-parietal network showed decreased connectivity in bilateral dorsolateral prefrontal and posterior parietal regions and increased connectivity in the left angular gyrus, left hippocampus, and right thalamus (Wolf et al., 2010). Weakened connectivity in the left fronto-parietal network in dyslexia, even after behavioral remediation, has been shown (Koyama et al., 2013). This further represents the dysfunction in both EF and reading networks in teenagers with dyslexia. Hyperactivation of the right inferior frontal gyrus in teenagers with dyslexia during a reading task predicted greater reading improvement 2 years later, showing that possibly greater activation in frontal areas contributes to better gain later on (Hoeft et al., 2007).

**Figure 3:**
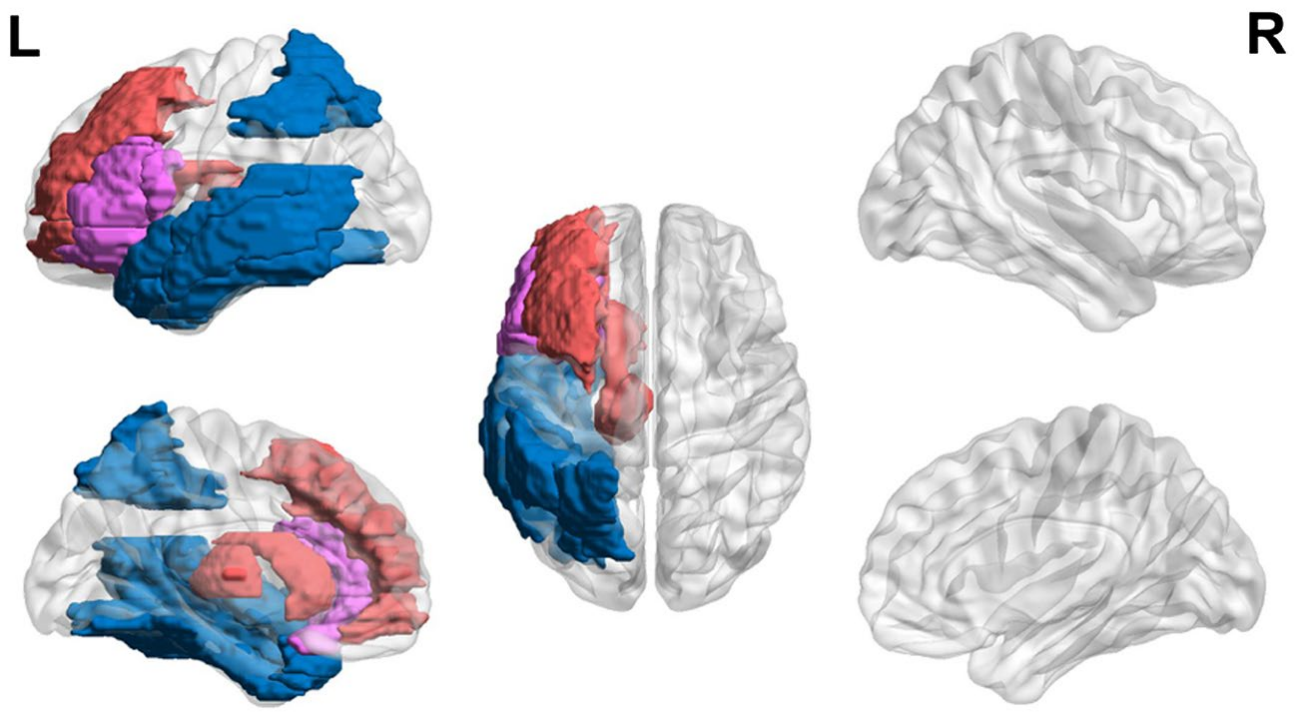
Neural circuit dysfunction related to reading circuits in teenagers (ages 13–21) with dyslexia. Decreased activation in the left inferior frontal gyrus (Wimmer et al., 2010; Steinbrink et al., 2012), left parietal lobule (Hoeft et al., 2007; Wimmer et al., 2010; Steinbrink et al., 2012), left fusiform gyrus (Hoeft et al., 2007; Desroches et al., 2010), and left temporal gyrus (Wimmer et al., 2010; Steinbrink et al., 2012) and increased activation in the left inferior frontal gyrus (Hoeft et al., 2007), left middle frontal gyrus (Hoeft et al., 2007), left caudate (Hoeft et al., 2007), and left thalamus (Hoeft et al., 2007) related to EF in children ages 13–21 at risk for dyslexia. Red represents areas of hyperactivity, blue areas of hypoactivity, and purple areas where papers have reported hyperactivity and hypoactivity. Data are presented in sagittal and axial views in the left and right hemispheres (L = left and R = right) over a glass brain. Regions were defined based on AAL atlas (Tzourio-Mazoyer et al., 2002). Image created using the BrainNet Viewer (Xia et al., 2013).

In sum, teenagers with dyslexia (ages 13–21 years) exhibit hyperactivation in EF areas (i.e., left inferior and middle frontal gyri and caudate) and the thalamus and hypoactivation in visual areas (i.e., left parietal and fusiform regions) and in posterior regions of the reading network. However, conflicting results of hypoactivation in the left and right inferior frontal region and hyperactivation in visual areas have been found as well. A general trend of hyperactivation in EF areas and hypoactivation in reading areas is found, but more studies need to be done to clarify conflicting results (Figure 3).

#### Neural Circuits Related to EF in Adults With Dyslexia (22 Years and Older)

**Figure 4:**
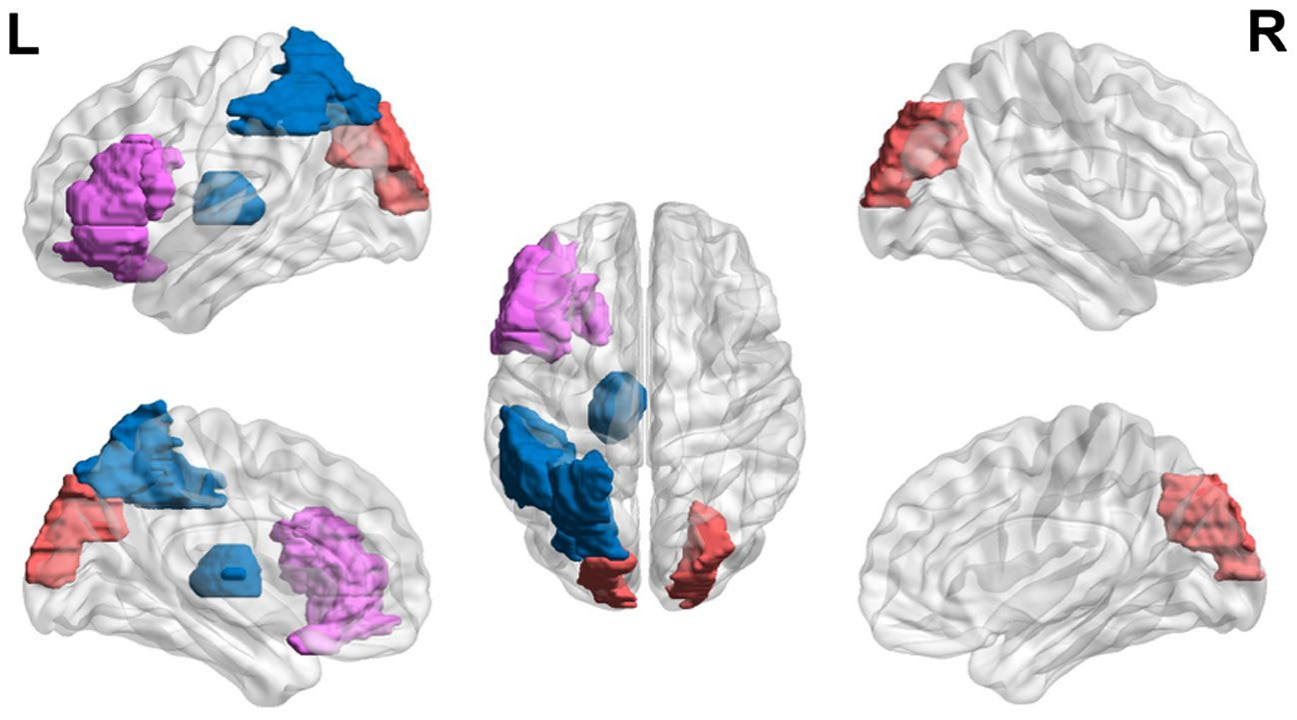
Neural circuit dysfunction related to reading circuits in adults (ages 22+) with dyslexia. Decreased activation in the left thalamus (Díaz et al., 2012), the left inferior frontal gyrus (Wimmer et al., 2010), and the left parietal lobule (Wimmer et al., 2010) and increased activation in the left inferior frontal gyrus (Karni et al., 2005) and bilateral superior occipital gyri (Wimmer et al., 2010) related to EF in adults ages 21 and up with dyslexia. Red represents areas of hyperactivity, blue areas of hypoactivity, and purple areas where papers have reported hyperactivity and hypoactivity. Data are presented in sagittal and axial views in the left and right hemispheres (L = left and R = right) over a glass brain. Regions were defined based on AAL atlas (Tzourio-Mazoyer et al., 2002). Image created using the BrainNet Viewer (Xia et al., 2013).

Lastly, studies have shown that adults with dyslexia may continue showing executive dysfunction during reading-related tasks (Figure 4). College students with dyslexia have been found to have lower error-related negativity amplitudes and later latencies in error responses compared to controls (Horowitz-Kraus and Breznitz, 2008, 2009).

Reading with time constraints shows neurobiological differences in regions related to vision and EF in adults with dyslexia (Karni et al., 2005). During fast reading, no significant activation differences were seen in the two groups. However, during slow non-word reading, hyperactivation in the left inferior frontal gyrus (involved in EF) and operculum was found, compared to the controls that showed activation in the visual areas (Karni et al., 2005). During a phonological task, hypoactivation in the auditory sensory thalamus (i.e., the medial geniculate body; Díaz et al., 2012), in a left ventral occipito-temporal region (related to orthography), a left inferior parietal region (related to attention) and a left inferior frontal region (Wimmer et al., 2010), and hyperactivation in visual occipital regions (Wimmer et al., 2010), and asymmetry in the inferior frontal gyrus (an EF area; Hernandez et al., 2013) was found in adults with dyslexia compared to controls.

Further, structural data supported these abnormalities in both EF and reading networks. Hinting at the importance of multisensory processing (Zeugin et al., 2017), decreased FA has been found in bilateral fronto-temporal and left temporo-parietal white matter regions (Steinbrink et al., 2008) and left middle and inferior temporal gyri and left arcuate fasciculus (Silani et al., 2005; Vandermosten et al., 2012). Decreased FA was found in bilateral temporo-parietal white matter regions in another sample of adults (Klingberg et al., 2000), and a decrease in FA in all four lobes has been seen (Richards et al., 2008).

Overall, the research supports that individuals with dyslexia may display difficulties in error detection and hypoactivation in reading-related and EF areas and hyperactivation in visual areas during phonological tasks even when reaching adulthood (ages 21 and up).

## Discussion

All age groups show dysfunction in neural circuits related to EF, showing that EF is involved in reading tasks and individuals with dyslexia experience a malfunction in these areas. Although the studies in the present review outline the abnormal activity of neural circuits related to EF and reading, another alternative is that abnormalities in brain areas related to EF could reflect compensation effects. While assessing very young children to determine the core neurobiological function of dyslexia are difficult, some studies have been done to determine when frontal dysfunction begins in children at risk for dyslexia. Delayed P100 and P200 peaks were seen during auditory stimulus presentation in 17-month-olds at risk for dyslexia (van Herten et al., 2008) and 3- to 5-year-old children with greater maternal fluency ability show increased functional connectivity between future reading networks and EF-related regions (Horowitz-Kraus et al., 2017), solidifying that EFs are developing quite early in correspondence with reading networks.

Overall, this review suggests a consistent phenomenon of lower behavioral EF abilities and alteration of neural circuits related to EF along development. These alterations were found in functional, structural, and network measures generated from MRI data as well as from EEG. These differences were also related to changes in visual processing regions [i.e., a left ventral occipito-temporal region, extrastriate regions, left parietal and fusiform regions, and the right and left occipital cortex (Shaywitz et al., 2002; Karni et al., 2005; Hoeft et al., 2007; Wimmer et al., 2010; Liu et al., 2012)].

One explanation of how EFs and reading development are connected can be explained by the inside-out and outside-in model. Emergent literacy involves both inside-out skills, such as phonological awareness and outside-in skills, such as conceptual knowledge (Whitehurst and Lonigan, 1998). EF is positioned in this model both as an inside-out factor which is part of the child’s essential abilities to learn language and reading and is involved in the outside-in factors contributing to reading by allowing the child to attend to stories, books, and other literacy material provided in the child’s development (Whitehurst et al., 1988). These data support the tight relations between nature (inside-out abilities) and nurture (outside-in factors, such as exposure to language, literacy, and parental reading) on neural circuits supporting future reading in the developing brain. See Table 2 for an overview of the neural circuits related to EF along development in children at risk and with dyslexia as well as in adults.

**Table 2:** Summary of studies assessing neurobiological changes during reading-related tasks along development.

Task	Children at risk (0–5)	Children with dyslexia (6–12)	Teenagers with dyslexia (13–21)	Adults with dyslexia (22+)
Narrative comprehension		Hyperactivation in the right superior frontal gyrus and right middle frontal gyrus (Horowitz-Kraus et al., 2016).		
		Hyperactivation in the left middle/superior temporal gyri and bilateral insula, right cingulate gyrus, right superior frontal gyrus, and right parietal lobe (Rimrodt et al., 2008).		
Phonology		Hyperactivation of the left extrastriate cortex and hypoactivation of the temporal and prefrontal cortex (Backes et al., 2002). Hypoactivation of left fusiform cortex (Desroches et al., 2010). Hyperactivity in the basal ganglia and hypoactivity in the left superior temporal gyrus (Kita et al., 2013). Hypoactivation in right visual and left occipito-temporal cortex and left inferior frontal gyrus (Liu et al., 2012). Hyperactivation in the left inferior frontal gyrus (Georgiewa et al., 2002). Absence of connectivity between lateral inferior frontal cortex and the anterior occipito-temporal cortex (Olulade et al., 2015). Aberrant activation of the parieto-temporal and occipito-temporal area (Shaywitz et al., 2002). Hyperactivation in the right cerebellum (van Ermingen-Marbach et al., 2013).	Dyslexic vs. age-matched – hyperactivation in the left inferior and middle frontal gyri, caudate, and thalamus. Age- and reading-matched – hypoactivation in left parietal and fusiform (Hoeft et al., 2007). Hypoactivation of left fusiform cortex (Desroches et al., 2010). Hypoactivation in posterior areas of reading network (Brambati et al., 2006). Hypoactivation in left ventral occipito-temporal region, a left inferior parietal region, and a left inferior frontal region (Steinbrink et al., 2012).	Hypoactivation in the left medial geniculate body (Díaz et al., 2012). Asymmetry in inferior frontal gyrus (Hernandez et al., 2013). Hypoactivation in left ventral occipito-temporal region, a left inferior parietal region, and a left inferior frontal region. Hyperactivation in visual occipital regions (Wimmer et al., 2010).
Reading		Hypoactivation left visual word form area and Wernicke’s area (Saralegui et al., 2014). Hyperactivation in the bilateral occipital cortex, inferior frontal regions, and inferior precentral gyrus (Seki et al., 2001).		Slow non-words – hyperactivation in the left inferior frontal gyrus and operculum (Karni et al., 2005).
Auditory stimuli	Event-related potentials mostly in right hemisphere compared to left hemisphere in controls (Lyytinen et al., 2004). No mismatch response (Zuijen et al., 2013). Delayed P100 and P200 peaks for standard auditory stimuli (van Herten et al., 2008). Hypoactivation in bilateral occipito-temporal and left temporo-parietal regions (Raschle et al., 2012).			
Lexical-semantic priming	Absent N200 response (von Koss Torkildsen et al., 2007).			

### Limitations

Some limitations exist in exploring if EF deficits in dyslexia occur before dysfunction in reading. It is important to note that some studies exploring EF and reading comprehension have found no correlation between the two, or only between certain EFs (Pennington et al., 1993; Nydén et al., 1999; Willcutt et al., 2001, 2005, 2010; Jeffries and Everatt, 2004; Cain, 2006; Bental and Tirosh, 2007; Altemeier et al., 2008; Tiffin-Richards et al., 2008; de Jong et al., 2009; Menghini et al., 2010; Gooch et al., 2011; Christopher et al., 2012; Peng et al., 2013; Varvara et al., 2014; Moura et al., 2015, 2017; Wang and Yang, 2015). However, the inconsistency in the research has been hypothesized to be due to several factors, including group classification difficulties, theoretical definition inconsistencies, and task impurity (Doyle et al., 2018). Lastly, although some developmental theories agree that speed of processing abilities is part of EF (Anderson and Reidy, 2012), this view is still under debate in the scientific community (Gordon et al., 2020).

### Conclusion

This review aimed to highlight the current behavioral and neurobiological atypicalities found in dyslexics along development in reading, highlighting EF regions and networks. As outlined earlier, individuals with dyslexia show altered brain activation and lower performance in higher-order EF tasks (such as WCST; Horowitz-Kraus, 2014, 2015; Kraus and Horowitz-Kraus, 2014), tasks which also involve reading abilities (e.g., Stroop task; Lazarus et al., 1984; Kelly et al., 1989; Everatt et al., 1997; Helland and Asbjørnsen, 2000; Reiter et al., 2005), or tasks that involve an auditory component (e.g., mismatch negativity; Lyytinen et al., 2004; van Herten et al., 2008; Raschle et al., 2012; Zuijen et al., 2013). One can postulate that one of the reasons for this altered performance is the tasks’ complexity, the involvement of reading, or the involvement of an auditory component. Although these tasks are well accepted in the literature as tasks examining EF (Axelrod et al., 1994; Romine et al., 2004; Carlson, 2005), another possibility is that the decreased performance in these tasks is a basic perceptual deficit rather than a deficit in EF. However, since vast literature suggested a decreased performance in additional, more basic EF tasks, such as inhibition (Brosnan et al., 2002; Gooch et al., 2014), speed of processing (Willcutt et al., 2005; Breznitz, 2006), and attention tasks (Gooch et al., 2014), additional prospective studies should be looking at this question in depth. Our review discussed the existence of conflicting results in reading networks in all age groups, namely, the left inferior frontal gyrus showing hyperactivity in some studies while displaying hypoactivity in others.

Overall, the reviewed evidence on dyslexia indicates that reading tasks are associated with hyperactive EF-related brain networks. Such a tight link between EF and reading disability highlights the importance of early assessment and intervention. Therefore, the inclusion of EF-specific neuro-behavioral testing in standard neuropsychological assessments will open new windows on the developmental profile of dyslexia which, in turn, will provide clinicians with early identification signatures for improved diagnosis and intervention. Such an early identification of children at risk for dyslexia will boost the implementation of interventions for EF and reading by strengthening or even altering some of the neurobiological and behavioral dysfunctions seen in dyslexia. Future research should aim to explore the conflicting results found in the literature to clarify the dyslexic profile. Specifically, it is crucial to explore specific EF networks activated alongside language networks during reading tasks to improve identification and intervention. Only after filling this needed research gap, it will be possible to design more comprehensive treatments for individuals with reading disabilities.

## Author Contributions

All authors listed have made a substantial, direct and intellectual contribution to the work and approved it for publication.

## Conflict of Interest

The authors declare that the research was conducted in the absence of any commercial or financial relationships that could be construed as a potential conflict of interest.

## Publisher’s Note

All claims expressed in this article are solely those of the authors and do not necessarily represent those of their affiliated organizations, or those of the publisher, the editors and the reviewers. Any product that may be evaluated in this article, or claim that may be made by its manufacturer, is not guaranteed or endorsed by the publisher.
